# An epigenetic timer regulates the transition from cell division to cell expansion during Arabidopsis petal organogenesis

**DOI:** 10.1371/journal.pgen.1011203

**Published:** 2024-03-05

**Authors:** Ruirui Huang, Vivian F. Irish

**Affiliations:** 1 Department of Molecular, Cellular and Developmental Biology, Yale University, New Haven, Connecticut, United States of America; 2 Department of Ecology and Evolutionary Biology, Yale University, New Haven, Connecticut, United States of America; Gregor Mendel Institute of Molecular Plant Biology, AUSTRIA

## Abstract

A number of studies have demonstrated that epigenetic factors regulate plant developmental timing in response to environmental changes. However, we still have an incomplete view of how epigenetic factors can regulate developmental events such as organogenesis, and the transition from cell division to cell expansion, in plants. The small number of cell types and the relatively simple developmental progression required to form the Arabidopsis petal makes it a good model to investigate the molecular mechanisms driving plant organogenesis. In this study, we investigated how the RABBIT EARS (RBE) transcriptional repressor maintains the downregulation of its downstream direct target, *TCP5*, long after RBE expression dissipates. We showed that RBE recruits the Groucho/Tup1-like corepressor TOPLESS (TPL) to repress *TCP5* transcription in petal primordia. This process involves multiple layers of changes such as remodeling of chromatin accessibility, alteration of RNA polymerase activity, and histone modifications, resulting in an epigenetic memory that is maintained through multiple cell divisions. This memory functions to maintain cell divisions during the early phase of petal development, and its attenuation in a cell division-dependent fashion later in development enables the transition from cell division to cell expansion. Overall, this study unveils a novel mechanism by which the memory of an epigenetic state, and its cell-cycle regulated decay, acts as a timer to precisely control organogenesis.

## Author summary

Epigenetic changes, such as histone modifications or DNA methylation, can modify gene expression without affecting the genetic code. Such alterations can persist for many cell generations, and often are induced by various environmental inputs in both plants and animals. By contrast, little is known of how intrinsic epigenetic changes can act to regulate the temporal progression of development. Here we showed that the expression of RABBIT EARS (RBE) represses the transcription of its target, *TCP5*, through inducing a variety of epigenetic changes at the *TCP5* locus. These changes dissipated over a period of days, allowing for eventual activation of *TCP5* expression and a concomitant shift from cell division to cell expansion during Arabidopsis petal development. The gradual loss of repressive epigenetic marks at the *TCP5* locus represents an internal timing mechanism to temporally control the development of the Arabidopsis petal.

## Introduction

In plants, organogenesis generally occurs via the successive processes of cell division followed by cell expansion [1]. The dynamics of this transition from cell division to cell expansion have been well documented in the Arabidopsis petal, providing an excellent model system with which to examine the molecular mechanisms driving this shift [2,3]. It has been previously suggested that this developmental shift may reflect the attenuation of chromatin-mediated silencing of genes whose expression is required to promote cell expansion and inhibit cell division [3].

In eukaryotes, chromatin is organized into subunits comprising the nucleosome, which consists of ∼147 bp DNA wrapped around a histone octamer containing a core (H3-H4)_2_ tetramer flanked by two H2A-H2B dimers [4]. Alterations in the organization of chromatin, caused by post-translational modifications of histone proteins or DNA methylation, can affect the accessibility of chromatin to the transcriptional machinery [5]. Histone modifications in particular can remodel chromatin structure by altering chromatin accessibility or by influencing the recruitment of effector proteins [6,7]. For example, histone acetylation allows the chromatin to “loosen” and allows for transcription factors and RNA polymerases to access the DNA [8]. By contrast, trimethylation of lysine 27 on histone H3 (H3K27me3), which is a repressive histone modification catalyzed by Polycomb-group (PcG) proteins that are present in all multicellular eukaryotes, has the opposite effect on transcription and is a repressive histone modification mark in plants [9].

As the key histone modifications acquired during development are inherited through multiple cell divisions, an ‘epigenetic memory’ is established that underlies the phenotypic stability of the differentiated cell state [10,11]. The maintenance of epigenetic states is key for defining cell and tissue-type identities in a number of contexts, and yet the lability of such states is also critical for cells to respond to extrinsic and intrinsic cues [12,13]. Transcription factors are able to reshape the chromatin landscape across regions they bind to, both through enabling the binding of other transcription factors and by direct recruitment of various histone modifiers [14–16].

Histone post-translational modifications include acetylation, methylation, ubiquitylation, phosphorylation and many other modifications [17]. Histone acetylation, catalyzed by histone acetyltransferases (HATs), mediates the de-condensation of chromatin while histone deacetylases (HDACs) induce chromatin compaction and repression of associated gene expression. Many HATs and HDACs are components of large chromatin remodeling complexes, which are recruited to gene promoters by DNA-bound proteins such as transcription factors [18]. For example, HDA19 interacts with the Groucho/Tup1-like TOPLESS (TPL) co-repressor as part of a larger complex, and is recruited by transcription factors containing the Ethylene-responsive element binding factor-associated Amphiphilic Repression (EAR) motif to repress transcription in plants [19–21].

The Arabidopsis *RABBIT EARS (RBE)* gene encodes a C2H2 zinc finger DNA binding motif and an LXLXLX type EAR motif and regulates several pathways required for normal petal development [2,22]. *RBE* is expressed in petal primordia during early stages of petal development and loss of function *rbe* mutants exhibit alterations in the initiation and development of petal primordia [2,22,23]. During the early phase of petal initiation, *RBE* regulates an *MIR164*-dependent pathway to control cell proliferation at petal primordium boundaries [24]. During petal development, RBE binds to the promoters of several TCP transcription factor genes to regulate their expression [3,25]. For example, RBE transcriptionally represses *TCP5* that in turn acts to inhibit the number and duration of cell divisions in the petal. The alleviation of the transcriptional repression of *TCP5* conferred by RBE results in the transition from cell division to post-mitotic expansion [3]. Interestingly, there is a considerable lag time of approximately six days between the downregulation of *RBE* expression [22,26] and the upregulation of *TCP5* expression, suggesting that this process may involve attenuation of chromatin-mediated silencing of *TCP5* expression [3,27].

Here we investigated the mechanisms by which RBE modulates the temporal expression of *TCP5*, and other genes involved in petal development. We showed that RBE physically interacts with the TPL-HDA19 corepressor complex through its EAR motif to negatively regulate petal epidermal cell expansion. We also showed that early in petal development, the transcriptional repression of *TCP5* is associated with lack of chromatin accessibility and a number of repressive histone modification marks. Furthermore, decreased chromatin accessibility close to the *TCP5* transcription start site was associated with a low occupancy of RNA polymerases, establishing a repressive state. Subsequent to the decay of the RBE protein, epigenetic ‘memory’ of the repressed state of *TCP5* was maintained, allowing for cell division to occur. The alleviation of this repressive memory allows for the activation of *TCP5* transcription and the concomitant transition from cell division to cell expansion. Additionally, we found that the diminution of repressive memory is cell division dependent, linking the rate of cell division to a timer controlling organogenesis.

## Results

### RBE physically interacts with the TPL-HDA19 corepressor complex

**Fig 1:**
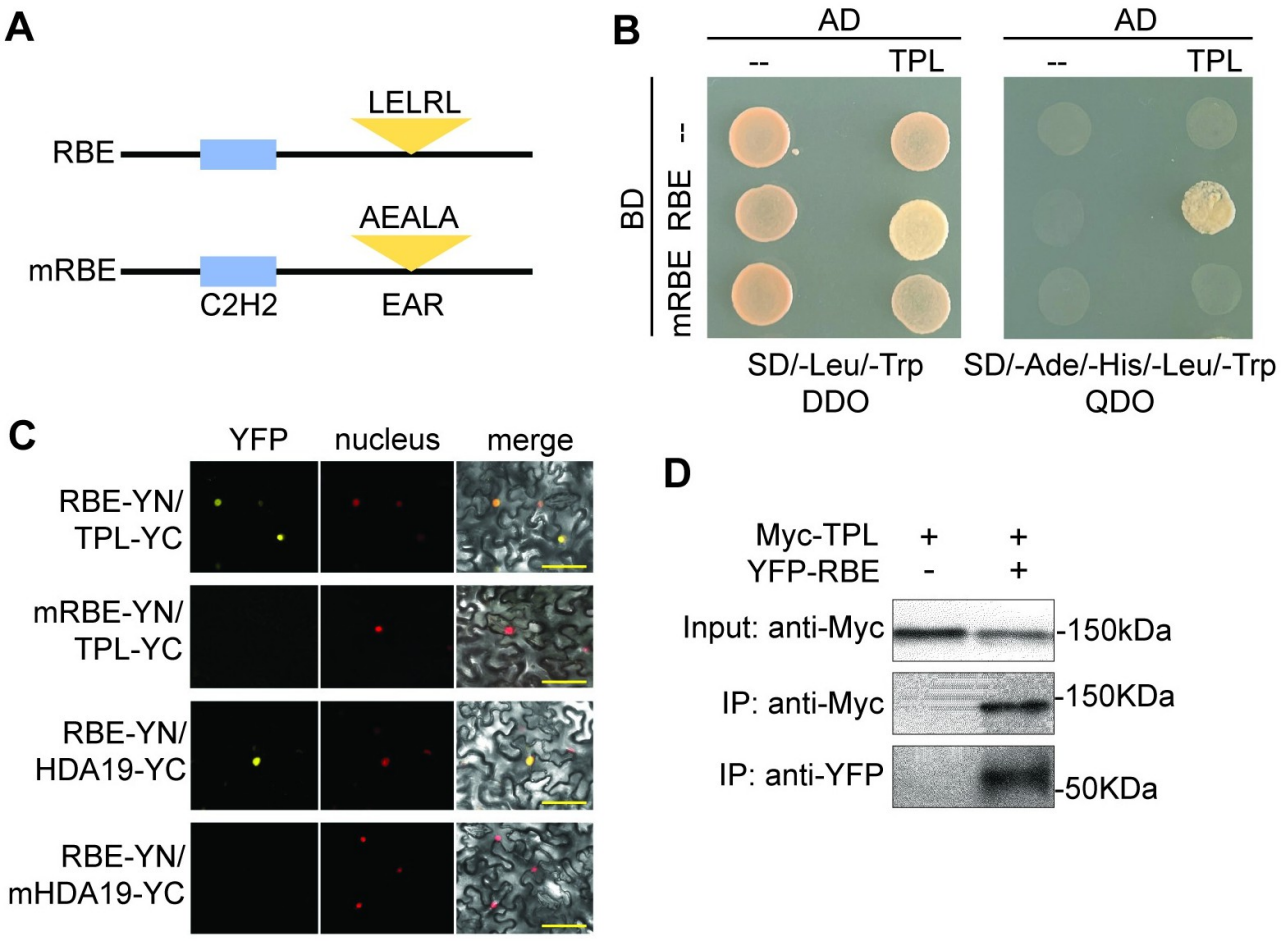
RBE physically associates with the TPL protein. (A) Diagram of native RBE protein and RBE with point mutations in the EAR motif (mRBE). Native and altered amino acid sequences are shown. (B) Yeast two-hybrid assays between RBE or mRBE with TOPLESS (TPL).—symbol indicates only the AD domain. Double Dropout (DDO) and Quadruple Dropout (QDO) were used as selective media. (C) Bimolecular fluorescence complementation assay (BiFC) to detect reconstitution of YFP fluorescence. YFP fluorescence shows interaction between RBE and TPL in the nuclei. Position of nuclei detected by fluorescence of H2B-mCherry. Panels (left to right): YFP; H2B-mCherry; merged. Scale bars, 50 um (D) Co-immunoprecipitation (Co-IP) of YFP-RBE and Myc-TPL in Agrobacterium-infiltrated leaves of *N*. *benthamiana*. Extracts were immuno-precipitated with anti-GFP antibody and detected using anti-GFP and anti-MYC antibodies. The experiment was repeated three times with similar results. IP, immunoprecipitation.

It has been shown that LxLxL type EAR motif-containing proteins mediate transcriptional repression through interaction with the Groucho/Tup1-like transcriptional cofactor TOPLESS (TPL) and with TPL-related (TPR) proteins [19,28,29]. To identify potential RBE-interacting cofactors, we used yeast two-hybrid assays and found that RBE physically interacted with the TPL protein. Mutation of the three conserved Leu residues of the EAR motif in RBE abolished the interactions between RBE and TPL proteins, indicating that RBE interacted with TPL through the EAR motif (Fig 1A and 1B). To confirm the interaction between RBE and the TPL *in planta*, we performed bimolecular fluorescence complementation (BiFC) assays, in which RBE and TPL were fused to amino- and carboxy-terminal moieties of the yellow fluorescent protein (YFPn and YFPc), respectively. Strong signals in the nuclei were observed when YFPn-RBE was co-transformed with YFPc-TPL (Fig 1C). These protein interactions were abrogated when there were point mutations in the EAR motif, indicating that the EAR motif is necessary for RBE-TPL interactions. RBE-TPL interactions *in planta* were further assessed by co-immunoprecipitation. We found that YFP-RBE was able to pull down Myc-TPL (Fig 1D), supporting the observations from yeast two-hybrid and BiFC assays that RBE physically interacts with TPL. We also examined the ability of TPR proteins to interact with RBE *in planta* using BiFC assays and found that TPR2, TPR3 and TPR4 all interacted with RBE in nuclei (S1 Fig), supporting the idea that RBE interacts with TPL/TPR proteins *in planta*.

TPL has been shown to recruit the histone deacetylase HDA19 to regulate transcription in plants [19]. To test whether RBE also interacts with HDA19, BiFC assays were carried out. Interactions between YFPn-RBE and YFPc-HDA19 were observed in nuclei, and these protein interactions were abolished when there were point mutations in the EAR motif of RBE, indicating that the RBE EAR motif is necessary for the recruitment of HDA19 (Fig 1C). Additionally, we also showed that the RBE protein interacts with TFL2/LHP1 and Lysine-Specific Demethylase 1 (LSD1)-like histone demethylases, LDL1 through its EAR motif (S2–S4 Figs).

### RBE recruits TPL through its EAR motif to regulate petal development

**Fig 2:**
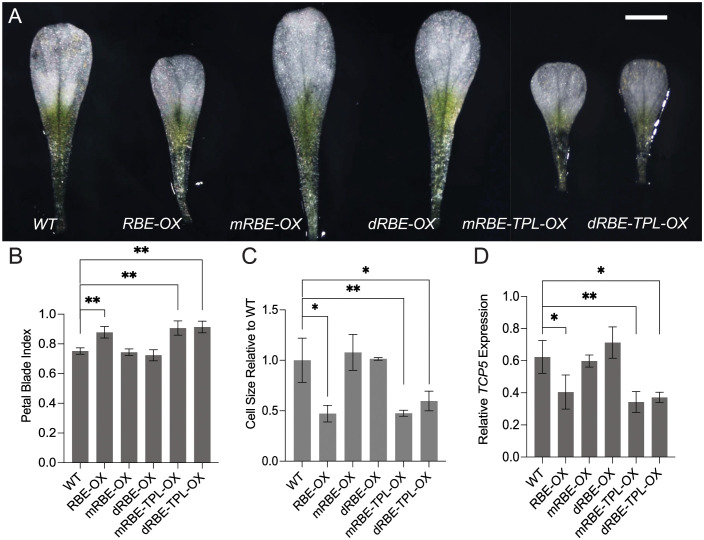
The EAR motif is necessary for RBE repressive effects during petal development. (A) Overexpression lines of *RBE*, *35S*::*RBE (RBE-OX)*, *35S*::*RBE* with an EAR motif mutation (*mRBE-OX*), *35S*::*RBE* with an EAR motif deletion (*dRBE-OX*), *TPL* fusion to *mRBE* (*mRBE-TPL-OX*) and *TPL* fusion to *dRBE (dRBE-TPL-OX*). Scale bar, 0.5 cm. (B) Quantification of petal blade index (width/length) of representative overexpression lines. Overexpression lines with more than 5-fold gene expression changes were quantified (S5A Fig). Error bars represent mean ± SD of four biological replicates. (C) Quantification of petal epidermal cell size of representative overexpression lines. Error bars represent mean ± SD of four biological replicates. (D) Relative levels of *TCP5* expression as assessed by qRT-PCR in seedlings of representative overexpression lines. *Tip41-like* was used as an internal control. Error bars represent mean ± SD of four biological replicates. *t*-test, **P<0.01, *P<0.05.

To functionally characterize the EAR motif of RBE, we generated transgenic lines overexpressing wild type *RBE (RBE-OX)*, *RBE* with the EAR motif mutated *(mRBE-OX)*, and *RBE* with the EAR motif deleted (*dRBE-OX)* (Figs 2A, S5A and S5B). The *RBE-OX* plants displayed petal phenotypes distinct from that of wild type, with the white petal blade being smaller and less well expanded (Figs 2A, 2B and S5B), which is consistent with the petal phenotype observed in plants with transient overexpression of RBE [3]. By contrast, the *mRBE-OX* and *dRBE-OX* lines had petals that were similar in size and shape to that of the wild type (Figs 2A, 2B and S5B). This indicates that the EAR motif is necessary for RBE function *in planta*.

To assess whether the fusion of TPL to mRBE or dRBE could rescue the mRBE or dRBE phenotypes, we generated transgenic plants overexpressing either an mRBE-TPL or dRBE-TPL fusion protein (Figs 2A and S5A). The fusion of TPL to the C terminus of mRBE-OX or dRBE-OX recapitulated the small petal phenotypes that were characteristic of the *RBE-OX* lines (Figs 2A, 2B and S5B), indicating that fusing the TPL protein to RBE complements the loss of EAR motif regulatory functions during petal development.

To examine whether the petal blade phenotypes were caused by defects in petal cell expansion or cell division, we quantified epidermal cell size in the distal blade region. *RBE-OX* petal blades showed a significant decrease in cell size compared with wild type, *mRBE-OX* or *dRBE-OX* (Fig 2C). By contrast, *mRBE-TPL-OX* and *dRBE-TPL-OX* petal blades displayed a smaller epidermal cell size phenotype similar to that of *RBE-OX*. Furthermore, the *RBE-OX* plants displayed curled and small leaves (S5C Fig). The *mRBE-TPL-OX* and *dRBE-TPL-OX* lines had similar leaf phenotypes as those of *RBE-OX*, whereas the *mRBE-OX* and *dRBE-OX* plants did not show any overt leaf phenotypes (S5C Fig). These data together support the idea that the overexpression of RBE activity represses cell expansion and RBE functions by recruiting TPL through the EAR motif to regulate petal development.

Since *TCP5* activity is required for petal cell expansion [3], we examined whether alteration of *TCP5* expression could explain the cell size defects in *RBE-OX*, *mRBE-TPL-OX* and *dRBE-TPL-OX* lines. Quantitative reverse transcription (qRT)-PCR showed that *TCP5* transcription levels were significantly downregulated in *RBE-OX*, *mRBE-TPL-OX* and *dRBE-TPL-OX* lines as compared with other transgenic lines and wild type plants (Fig 2D). This further suggests that RBE recruits TPL through the EAR motif to regulate *TCP5* transcription and in turn to regulate petal epidermal cell expansion.

### RBE cooperates with TPL and HDA19 to repress *TCP5* during petal development

**Fig 3:**
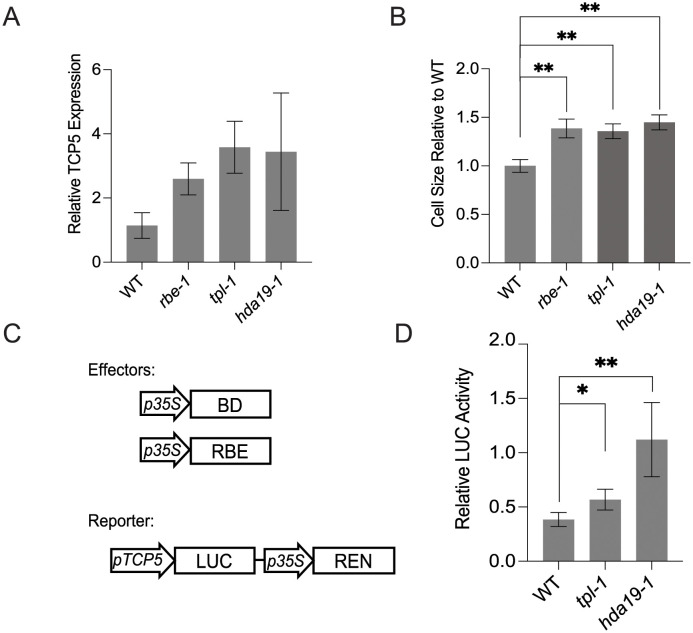
TPL and HDA19 repress *TCP5* expression during petal development. (A) Relative levels of *TCP5* expression as assessed by qRT-PCR in wild type, *rbe-1*, *tpl-1*, *hda19-1*. Error bars represent mean ± SEM of three biological replicates; the differences were not statistically significant. *ACT2* was used as the internal control. (B) The petal cell size of different genotypes. *t*-test, error bars represent mean ± SD of three biological replicates. (C, D) The repressive activity conferred by RBE in different genotypes; effectors and reporter diagrammed in (C), and relative activity (RBE/BD) shown in (D). Error bars represent mean ± SD of four biological replicates. *t*-test, **P< 0.01, *P <0.05.

The molecular function of the RBE-TPL-HDA19 interaction was investigated first by qRT-PCR, and the results showed that expression of *TCP5* was upregulated in *tpl-1* and *hda19-1* as compared with that of wild type plants (Fig 3A). Because *TCP5* functions to promote petal cell expansion, we therefore quantified the petal blade epidermal cell size of *tpl-1* and *hda19-1* plants to investigate whether TPL or HDA19 regulated petal cell expansion. For each mutant, the cell size was significantly larger than that of wild type, which was consistent with our qRT-PCR data (Fig 3B).

To investigate whether RBE interacts with the TPL-HDA19 complex to repress *TCP5* transcription, we conducted dual luciferase assays (Fig 3C). In wild type protoplasts, an RBE effector conferred strong repressive effects on the *TCP5* promoter. However, in protoplasts containing the *tpl-1* mutation, the repressive effects of RBE were attenuated. Furthermore, in *hda19-1* protoplasts the repressive effects of RBE were completely abrogated. These data together indicate that TPL and HDA19 are necessary for RBE-mediated repression of the transcription of its target gene *TCP5*.

**Fig 4:**
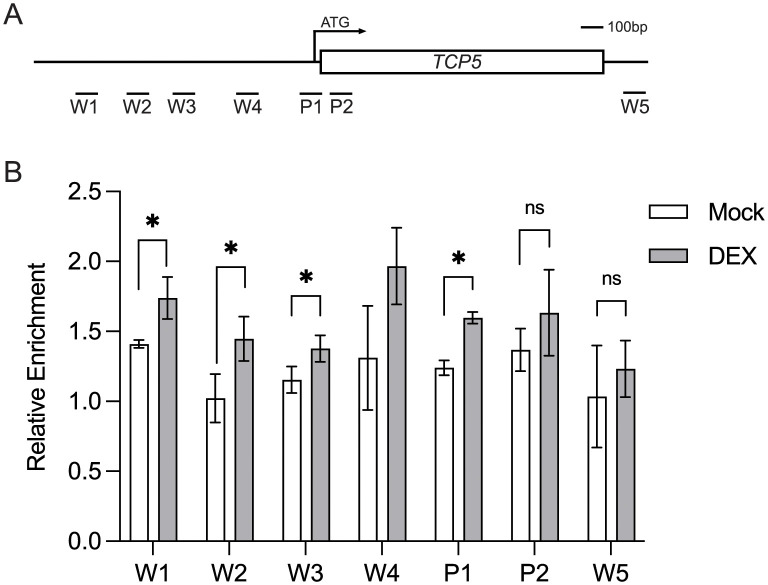
TPL binds to *TCP5* promoter regions. (A) Schematic diagram showing the *TCP5* locus and the genomic regions used for ChIP assays in (B). (B) ChIP assays using *35S*::*GR-RBE TPLp*::*TPL-HA* one week old seedlings after 16h DEX treatment. *Mu-like* transposon served as the negative control and its value was set to 1. Error bars represent mean ± SD of three biological replicates. **P< 0.01, *P <0.05.

To further examine whether TPL, in the presence of RBE, could bind to the *TCP5* promoter region to regulate its transcription, we performed chromatin-immunoprecipitation (ChIP) assays using dexamethasone (DEX) inducible *35S*::*GR-RBE* plants containing a *TPLp*::*TPL-HA* transgene (Fig 4). The relative enrichment of specific *TCP5* promoter sequences were tested by qRT-PCR. The enrichment of TPL on *TCP5* promoter regions was detected using and anti-HA antibody with significant enrichment observed in the presence of RBE, at the *TCP5* promoter regions W1, W2, W3 and P1 (Fig 4A and 4B). Consistent with this, the TPL homologue-TPR1 acts as a transcriptional corepressor and preferentially binds to regions around 1kb upstream of the TSS [30]. We also carried out ChIP assays using a *35S*::*Myc-HDA19* transgene. The relative enrichment of HDA19 on all sites with the exception of W5 suggests that HDA19 may bind to *TCP5* in the absence of RBE (S6 Fig). In addition, we did not observe significant enrichment of HDA19 across *TCP5* in the DEX-treated samples as compared to mock (S6 Fig). This may reflect the requirement of other, unknown proteins in facilitating binding of HDA19 to TPL in the presence of RBE. Additionally, previous ChIP experiments have demonstrated that the RBE protein is significantly enriched at the W1 and W3 regions [3]. These data together suggest RBE recruits the TPL chromatin remodeler to the *TCP5* promoter, to regulate its transcription. Furthermore, the significant enrichment of RBE and TPL together at the W1 region suggests that this promoter region is particularly important in the epigenetic regulation of *TCP5* expression.

### RBE induces chromatin remodeling to regulate *TCP5* transcription

**Fig 5:**
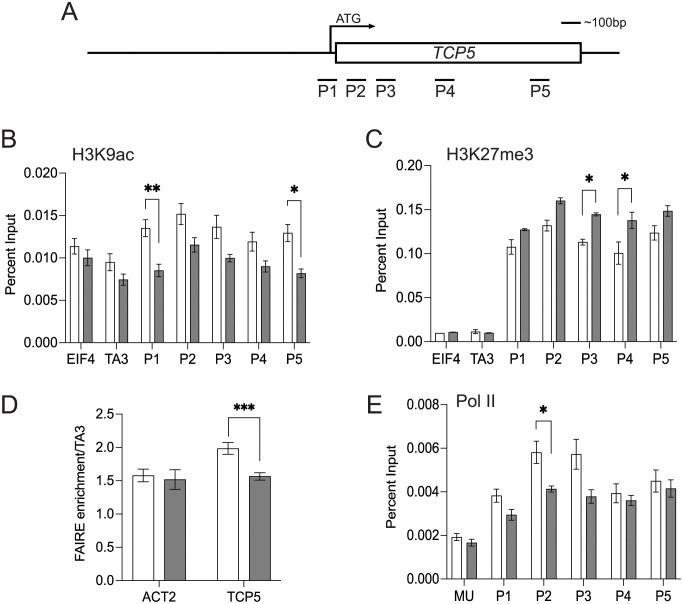
*TCP5* chromatin dynamics after RBE transient overexpression. The regions used for ChIP assays are diagrammed in Fig 4A. (A) H3K9ac analysis by ChIP assays using inflorescences 16h after a single 10μM DEX (grey bars) or mock (empty bars) treatment. The *TA3* retrotransposon and *EIF4* genes were used for negative controls. Error bars represent mean ± SEM of four biological replicates. (B) H3K27me3 analysis by ChIP assays using inflorescences 16h after a single 10μM DEX (grey bars) or mock (empty bars) treatment. Error bars represent mean ± SEM of two biological replicates. (C) DNA accessibility at the transcription start site (P1) of *TCP5* assayed by FAIRE 16h after a single 10μM DEX (grey bars) or mock (empty bars) treatment. The *TA3* retrotransposon was used a negative control and was set to 1. Error bars represent mean ± SD of four biological replicates. (D) ChIP assay for RNA Pol II binding using inflorescences 16h after a single 10μM DEX (grey bars) or mock (empty bars) treatment. The *Mu-like* transposon served as a negative control. Error bars represent mean ± SEM of four biological replicates. *t*-test, **P< 0.01, *P <0.05.

To investigate whether the transcriptional repression of *TCP5* is correlated with TPL-mediated chromatin remodeling, epigenetic modification changes were examined after transient overexpression of *RBE* using the *35S*::*GR-RBE* system. Because RBE appears to recruit the TPL-HDA19 complex to regulate *TCP5*, a decrease in histone acetylation levels across the *TCP5* locus would be predicted to occur after DEX treatment. As expected, *TCP5* transcription displayed significant downregulation 16h after DEX treatment (S7 Fig), which is consistent with the observation that *TCP5* transcription is significantly downregulated 4h after DEX treatment in the *35S*::*GR-RBE* system [3]. This also implies that the institution of the repressive state via the induction of RBE expression is not cell-cycle dependent. We first tested for the presence of the HDA19-regulated histone acetylation mark H3K9ac across the *TCP5* genomic locus by ChIP-qPCR 16h after DEX treatment. Genomic-wide profiling of H3 acetylation has shown that H3K9ac peaks are centered close to transcriptional start sites (TSS) [31,32]. Consistent with this observation, we detected a significant H3K9ac decrease in the P1 region proximal to *TCP5* TSS, as well as the end of coding region (P5) after 16h DEX treatment as compared with the mock treatment (Figs 4A and 5A). Similarly, TPL-mediated chromatin remodeling has been shown to be associated with dynamic changes in H3K27me3 status as catalyzed by PcG proteins [33,34]. Addtionally, our protein interaction data also indicates that RBE physically interacts with LHP1 (S2 Fig). We observed a significant increase of H3K27me3 across transcribed regions (P3 and P4) of *TCP5* in DEX treated samples as compared to mock (Figs 4A and 5B), which is consistent with H3K27me3 enriched profiles across *TCP5* from ChIP-seq data [9]. These results support the idea that RBE triggers transcriptional repression of *TCP5* by regulating TPL-mediated histone modifications.

It has been reported that the local state of chromatin compaction around transcription start sites determine transcription levels [35]. To comprehensively understand the chromatin remodeling events occurring at the *TCP5* locus induced by RBE, we tested DNA accessibility changes at known DNase I hypersensitive sites [36]. We performed formaldehyde assisted identification of regulatory elements (FAIRE) followed by quantitative PCR, a method that detects accessible (nucleosome-depleted) genomic regions [37]. In 16h DEX treated samples as compared to mock treated controls, we observed a significant decrease in chromatin accessibility at the P1 region close to the transcription start site of *TCP5* but not at the *ACT2* locus (5C). Previous reports have indicated that there is a direct correlation between chromatin accessibility and RNA polymerase binding around TSSs [36,38]. To test whether RBE remodels chromatin and influences RNA polymerase binding, we performed ChIP assays to examine RNA polymerase II (Pol II) enrichment at the *TCP5* locus (5D). In 16h DEX treated samples as compared to mock treated controls, we observed a significant decrease of Pol II enrichment immediately after the TSS site, which is consistent with observations at other loci [36]. This is also consistent with the finding that H3K9ac close to TSS promotes Pol II pause release to switch from transcription initiation to elongation [39]. These data together indicated that RBE induces changes in histone modification patterns and chromatin remodeling events at the *TCP*5 locus that presumably contribute to its transcriptional repression.

### Transient RBE overexpression induces an ‘epigenetic memory’ to maintain downregulation of *TCP5* transcription

**Fig 6:**
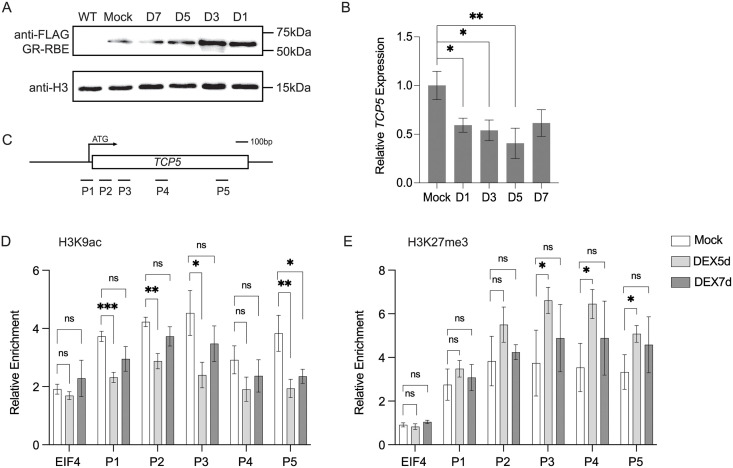
RBE transient expression induces the transcriptional delay of *TCP5* expression. (A) Nuclear accumulation of the GR-RBE fusion protein after a single 10μM DEX treatment. Inflorescence tissues were collected at 1d, 3d, 5d and 7d. The GR-RBE fusion protein and histone H3 as nuclear internal control were detected by immunoblotting. (B) Relative levels of *TCP5* expression as assessed by qRT-PCR in floral tissues collected at 1d, 3d, 5d and 7d. Error bars represent mean ± SD of at least three biological replicates. The *TCP5* genomic regions used for ChIP assays in (C) and (D) are diagrammed in Fig 4A. (C) H3K9ac analysis by ChIP assays using inflorescences 5d (light grey bars) and 7d (dark grey bars) after a single 10μM DEX treatment or mock treatment (empty bars). Relative enrichment indicates percent input normalized to that of *TA3*. Error bars represent mean ± SD of three biological replicates. (D) H3K27me3 analysis by ChIP assays using inflorescences 5d (light grey bars) and 7d (dark grey bars) after a single 10μM DEX treatment (grey bars) or mock treatment (empty bars). Relative enrichment indicates percent input normalized to that of *TA3*. Error bars represent mean ± SD of three biological replicates. *t*-test, **P< 0.01, *P <0.05.

To investigate the temporal parameters of RBE function we first tested the nuclear RBE protein decay rate in *35S*::*GR-RBE* lines. Protein extracts were generated from nuclei isolated from inflorescence tissues that were either DEX- or mock treated. Plant tissues were collected at 1d, 3d, 5d, and 7d after treatment. The immunoblot results showed that GR-RBE protein strongly accumulated in nuclear extracts of DEX treated plants 1d and 3d after the treatment (Figs 6A and S8). By 5d, the GR-RBE protein levels detected in nuclear extracts of DEX treated tissues were decreased to a level similar to that of mock treated tissues, suggesting that the GR-RBE protein that had entered the nucleus in response to the DEX treatment had been degraded or had undergone nuclear export. We next performed qRT-PCR to test *TCP5* transcription levels in mock treated or DEX treated inflorescence tissues. The *TCP5* transcription level was significantly downregulated 1d, 3d, and 5d after the DEX treatment compared to the mock treatment, and this downregulation was maintained through 7d (Fig 6B). This suggests that *TCP5* transcription was still repressed for several days after the over-expressed nuclear GR-RBE protein had decreased to the mock treated level.

To further investigate the temporal parameters of chromatin remodeling associated with the downregulation of *TCP5* expression when induced by transient RBE overexpression, we conducted a timecourse of ChIP assays, focusing on day 5 and day 7 after DEX treatment, to test the histone modification levels across the *TCP5* locus. The H3K9ac levels close to the TSS region of *TCP5* were significantly lower at P1, P2 and P3 in inflorescence tissues 5d after DEX treatment as compared to that of mock treated tissues, and the decreased acetylation level was reversed 7 days after DEX treatment (Fig 6C), which is consistent with the observation that H3K9ac close to the transcription start site promotes transcription initiation to elongation [39]. Interestingly, H3K9ac at P5, which is close to the Transcription End Site (TES), is also significantly decreased 16h, 5d after DEX treatment but it is not reversed 7 day after DEX treatment (Figs 5A and 6C). This indicates that H3K9ac close to the TSS might have different regulatory functions from that near the TES. Concomitantly, H3K27me3 levels across transcribed regions at P3, P4 and P5 of *TCP5* were significantly higher in inflorescence tissues 5d after DEX treatment as compared with that of mock treated tissues (Fig 6D). This supports our hypothesis that transient overexpression of RBE results in transcriptional delay of *TCP5* by inducing chromatin remodeling that is maintained as a repressive epigenetic memory over a number of days.

### Induction of *TCP5* transcription is cell division-dependent

**Fig 7:**
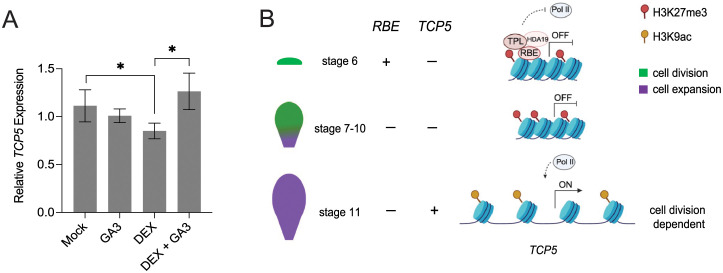
Induction of *TCP5* is cell division dependent and the temporal regulatory model. (A) Relative levels of *TCP5* expression as assessed by qRT-PCR in floral tissues collected 5d after mock treatment, 50μM gibberellic acid 3 (GA_3_) alone, 10μM DEX alone, and 10μM DEX with GA_3_. *Tip41-like* served as the internal control. Error bars represent mean ± SD of three biological replicates. *t*-test, **P< 0.01, *P <0.05. (B) A model of epigenetic timing mechanisms induced by RBE to regulate *TCP5*.

It has been shown that Polycomb protein–mediated repressive memory is diluted out after several rounds of DNA replication [40,41]. To assess the mechanism of the timed removal of repressive memory on *TCP5*, the phytohormone gibberellin (GA_3_), which has been shown to accelerate cell cycle progression [41], was applied separately and in combination with DEX to treat *35S*::*GR-RBE* inflorescences [42,43]. Five days after treatment, qRT-PCR was performed to assess the levels of *TCP5* transcription in treated and control tissues (Fig 7A). As expected, *TCP5* transcription was significantly downregulated 5d after DEX treatment; however, the addition of GA_3_ alleviated the repression of *TCP5* expression induced by DEX treatment (Fig 7A). There was no significant effect on *TCP5* transcription after GA_3_ treatment as compared with the mock treatment, indicating that GA_3_ alone had no significant effect on this process in our floral experimental system. Thus, replication-dependent alleviation of transcriptional repression is required for temporal regulation of *TCP5* expression during petal organogenesis.

## Discussion

Particular chromatin states can be maintained through a variety of enzymatic processes that act to copy histone and other epigenetic modifications to newly synthesized chromatin after DNA replication [44]. These dynamic processes can maintain a chromatin state in a stable manner through many cell generations, creating an ‘epigenetic memory’. Just as importantly, though, is the ability for a cell to alter its state to allow for changes in change expression; mechanisms that allow for a temporal shift in an epigenetic state, or ‘forgetting the memory’, are not as well understood. In this study, we have shown that the transient expression of the RBE transcriptional repressor can trigger epigenetic changes and repress transcription at a target locus, *TCP5*, and that the memory of this state is gradually ‘forgotten’ via cell division to allow for the expression of *TCP5*. We propose that in this genetic network, the process of cell division acts as an internal developmental timer to promote the shift in from cell division to cell expansion during petal organogenesis, a process controlled by *TCP5* [3].

We first demonstrated that RBE physically interacts with TPL-HDA19 complex proteins through a series of protein interaction assays. This is in line with several findings that EAR motif containing proteins genetically and physically interact with TPL and histone deacetylases to modulate epigenetic regulation of gene expression [19,28,29]. Recent reports indicate that the EAR motif could act as a docking point for the TPL-HDAC and PRC2 complexes which in turn can result in chromatin remodeling at a target locus by increasing H3K27me3 and decreasing H3ac levels [34,35,40]. For example, the Arabidopsis KNUCKLES (KNU) gene product contains an EAR motif, and functions to silence *WUSCHEL (WUS)* by recruiting the PCR2 protein FIE to deposit H3K27me3 marks [45]. Furthermore, SUPERMAN, an Arabidopsis gene product necessary for floral patterning, directly interacts with CLF, a catalytic subunit of the PRC2 complex, and TFL2/LHP1, which is known to interact with PcG proteins, through its EAR motif [46]. We also showed that RBE interacts with TFL2/LHP1through its EAR motif, which is consistent with our observation of H3K27me3 changes induced by RBE.

Additionally, we found RBE interacted with the Lysine-Specific Demethylase 1 (LSD1)-like histone demethylases, LDL1. Thus, our data supports a model in which RBE recruits a variety of histone modifiers to deposit multiple layers of histone modifications to induce epigenetic silencing at the *TCP5* locus (Fig 7B).

Patterns of histone modifications must be re-established to replicate the epigenetic status during cell division [47,48]. This process involves nucleosome remodeling, incorporation of histone variants and restoration of marks on DNA and histones [49]. The incorporation of various histone variants into nucleosomes has a marked impact on local chromatin structure and dynamics in plants [4,42]. For example, the histone variant H2A.Z has been linked to multiple biological process including flowering and cellular response including transcriptional activation and repression in plants [4,50]. A recent report indicating that the histone variant H2A.W cooperates with chromatin modifications to maintain transcriptional silencing of transposons in *Arabidopsis* [51].

We suspect that RBE induces deacetylation and compacts the chromatin close to the *TCP5* transcription start site first, followed by the deposition of the repressive histone modification mark H3K27me3 written by PcG proteins. There is precedent for sequential epigenetic modifications inducing a repressive state; for instance, KNU has been shown to first alter H3 acetylation, chromatin accessibility and Pol II binding at the *WUS* locus, whereas significant changes of H3K27me3 at *WUS* were observed only several hours later [45].

We have shown that the repressive memory induced by RBE is pivotal for continuous silencing of *TCP5* so as to maintain cell divisions through early stages of petal development. Unlike the relative stability of many epigenetic states, we have shown that after approximately seven days, *TCP5* transcription is restored and that then promotes cell expansion. This switch from repression to activation of *TCP5* depends on cell division as a timer, and as we have shown that by altering rate of cell division, the timing of *TCP5* transcriptional activation can be altered. We can envision several possibilities as to how silenced chromatin be remodeled to active chromatin in this developmental context. One possibility is that there is a class of pioneer transcription factors that can access their cognate binding motifs in closed chromatin to reprogram the chromatin state. For example, the pioneer transcription factor LEAFY (LFY) can contact its target gene *AP1* in compacted chromatin to reprogram floral cell fate by displacing the H1 linker histone and recruiting chromatin remodelers to promote chromatin accessibility [52]. This possibility would imply that an as yet unknown pioneer transcription factor is activated and binds to the *TCP5* promoter region to reactivate *TCP5* transcription at late stages of petal development.

Another non-mutually exclusive possibility is that repressive histone modification marks such as H3K27me3 could be diluted with the progression of DNA replication [40,41]. In plants, olomoucine which block cell cycle progression at the G1-S and G2-M phases, extends the timing of occupancy of repressive histone modification marks, while the application of phytohormone gibberellic acid 3 (GA_3_) promoting cell division accelerates the dilution of repressive marks [39,48,49]. We have demonstrated that the alleviation of transcriptional repression *TCP5* is dependent on cell division (Fig 7A). A corollary to this observation is that it implies that the mechanism to reinstall H3K27me3 after DNA replication, which depends on mono-methylation of histone 3.1 as well as binding of PcG proteins to the replication fork [48,49], is either non-functional or actively disrupted at the *TCP5* locus. Furthermore, mechanisms to actively remove PcG proteins during cell division have been described and can function as developmental timers [41]. As a consequence, it may be possible to manipulate the epigenome to modulate its response to DNA-replication dependent timing and to influence plant growth and development.

## Materials and methods

### Plant materials and growth conditions

*Arabidopsis thaliana* Columbia (Col-0) and Landsberg *erecta* (*Ler*) were used as wild types. The *rbe-1* [22], *tpl-1* [53] and *hda19-1* [54] mutants are in *Ler* background. The transgenic lines *TPLP*:: *TPL-HA* [55], *35S*::*10xMyc-HDA19* and *35S*::*GR-RBE* [24] are in *Ler* background. To genotype plants for these mutations, DNA was extracted and used as a template in PCR reactions with primers given in S1 Table. Seeds were sterilized and germinated on ½ Murashige and Skoog (MS) agar medium supplemented with 1% sucrose. Seedlings were transplanted at one week into a mix with two parts Vermiculite to one part of Fafard soil mix. Plant were grown under long day conditions (16h light/8 h dark) at 22°C.

### Yeast two hybrid assays

Yeast two hybrid (Y2H) assays were performed using the ProQuest expression vectors (Invitrogen) and Matchmaker Gold Yeast Two-Hybrid System products (TaKaRa). Full length coding sequences were PCR generated from *Arabidopsis* Col-0 cDNA using the Phusion DNA polymerase system and cloned into the PDONR207 vector using BP Clonase (Invitrogen). The Gateway compatible vectors pGADT7 (prey) and pGBKT7-GW (bait) were used as backbones [56]. Conserved leucines within the EAR motif of RBE were converted into alanines (**CTT**GAG **CTA** AGG **CTA** to **gcT**GAG **gcA**AGG **gcA**) by site directed mutagenesis. Bait and prey plasmids were obtained by LR reaction (Gateway) and co-transformed into the Y2H Gold yeast strain. SD-Leu-Trp agar plates (DDO) were used to select yeast harboring the bait and prey plasmids. SD-Leu-Trp-His agar plates (QDO) were used to analyze protein interactions. Experiments were repeated three times with similar results. Primers used are listed in S2 Table.

### Bimolecular fluorescence complementation assays

The Gateway compatible BiFC system with pEarleyGate201-YC and pEarleyGate202-YN as backbones was used as previously described [57]. The coding sequence without stop codon was amplified from plasmids used for Y2H and cloned into pDNOR207 using BP clonase (Invitrogen). RBE and mRBE were cloned into pEarleyGate202-YN, TPL and HDA19 were cloned into pEarleyGate201-YC through LR reaction. The resulting plasmids were transformed into *Agrobacterium* strain GV2260 by freeze shock. Subsequently, pairs of combinations were co-infiltrated into the 4 week old *N*. *benthamiana* leaves through *Agrobacterium* infiltration. P19 was used to inhibit transgenic silencing while H2B-RFP was used to visualize the nuclei [58]. The infiltrated leaves were dissected and imaged using a Zeiss LSM510 confocal laser scanning microscope 48h after infiltration. Results were verified in at least three repeats.

### Quantitative RT-PCR

To test *TCP5* expression in *rbe-1*, *tpl-1*, *hda19-1* mutants, total RNA was isolated from unopened floral buds from stage 1 to stage 12, along with the inflorescence meristem by using the Direct-zol RNA MiniPrep kit (ZYMO Research) with DNase I treatment to remove any contaminating genomic DNA. To test *TCP5* expression levels in multiple transgenic lines of RBE-OX variants, seedlings were used. Total RNA (1μg) was used for reverse transcription using an iScript cDNA synthesis kit (BioRad). qPCR was performed in a CFX96 Real time system using iQ SYBR Green SuperMix (Bio-Rad). The primers used for qRT-PCR are listed in S3 Table. Fold change and error bars were calculated from at least three biological replicates with three technical replicates.

### Plasmid constructions and transgenic plants

For construction of *35S*::*RBE*, *35S*::*mRBE* and *35S*::*dRBE*, pDONR-RBE, pDONR-mRBE and pDONR-dRBE were cloned into the binary vector pGWB521 by LR reaction. To make the mRBE-TPL and dRBE-TPL fusion proteins, the TPL coding sequence with stop codon was cloned into the PstI and NotI sites of the pRS300 vector [59]. mRBE and dRBE coding sequences without the stop codon were then cloned into the SalI and PstI sites of pRS300-TPL. RBE forward primer and TPL reverse primer were used to amplify the mRBE-TPL and dRBE-TPL fusion gene sequence from pRS300-mRBE-TPL and pRS300-dRBE-TPL. All regions corresponding to the transgene were cloned into the pDONR207 vector and then transferred to the PGWB521 vector by using the Gateway system (Invitrogen). To generate *35S*::*10xMyc-HDA19*, the *HDA19* coding sequence with stop codon was first amplified and cloned into pDONR207 vector by BP reaction and then transferred to PGWB521 vector by LR reaction. Vectors were transformed into *Agrobacterium* strain GV3101 and were selected with Spectinomycin. The resulting plasmids were transformed into *Ler* using the floral dip method and transgenic plants were selected using Hygromycin B [60].

### Phenotypic and kinematic analyses

To characterize and quantify petal phenotypes of transgenic overexpression lines, four representative F1 transgenic lines with approximate 5~20 fold change of *RBE* transcript levels as compared with *Ler* plants were selected for phenotypic quantification and gene expression analysis. For cell size measurements, petals were cleared with 70% ethanol and images of adaxial epidermal cells were taken using a Zeiss Axiophot microscope. Petal cell size was measured on the adaxial upper half of the blade. Three representative flowers from three plants for each genotype were examined. Average cell sizes were calculated from the number of cells per unit area in ImageJ as described previously [3].

### Co-Immunoprecipitation assay

Co-IP assays were performed as previously described [58]. Full-length coding sequences including the stop codon were amplified and cloned into PGWB521 or pEarleyGate104 to yield Myc-TPL or YFP-RBE, respectively. These constructs were transformed into Agrobacterium strain GV2260. Combinations of constructs together with P19 were co-infiltrated into four-week-old *N*. *benthamiana* leaves. After 60 hours of growth, the leaves were chopped and ground into fine powder in liquid nitrogen. Proteins were purified by suspending the powder into an ice-cold extraction buffer and followed by four rounds of centrifugation until the supernatant was clear. 50 μL of supernatant was boiled to use as input and the remaining supernatant was incubated with 30 μL GFP-trap agarose beads with gentle rotation overnight at 4°C. The immunoprecipitates were washed 4 times and protein complexes were released with SDS loading buffer. Proteins were detected by Western-blotting using anti-Myc (Sigma, C3956) and anti-GFP (ab290, Abcam). Similar results were obtained from three biological replicates.

### Analysis of GR-RBE accumulation

Inflorescence tissue was collected from *35S*::*GR-RBE* plants at different time points after treatment with dexamethasone-containing or mock solution. Nuclear protein extraction was carried out as in [61]. The tissues were immediately immersed into liquid nitrogen and 0.5 g of inflorescence tissue was ground into powder in liquid nitrogen. Cells were resuspended in 2 ml lysis buffer (20 mM Tris-HCl pH 7.4, 25% glycerol, 20 mM KCl, 2 mM EDTA, 2.5 mM MgCl_2_, 250 mM sucrose, 1 mM DTT, 1 of Roche protease inhibitor cocktail) and sequentially filtered through 100-μm and 40-μm cell strainers to remove cell debris. Flow-through was taken as the total lysate. Nuclei were pelleted by centrifugation (1,500g, 15 min, 4°C) and the supernatant was taken as the cytosol fraction. Nuclei were then washed 4 times in nuclei resuspension buffer (20 mM Tris-HCl pH 7.4, 25% glycerol, 250 mM NaCl, 2.5 mM MgCl_2_, 0.15% Triton X-100, 1 mM DTT and 1 of Roche protease inhibitor cocktail). After washing, nuclei were resuspended in PBS as the nuclear fraction. Proteins were detected by Western-blotting using anti-Flag (Sigma, A8592) and anti-H3 (ab1791, Abcam). Similar results were obtained from three biological replicates. The protein intensity was quantified by ImageJ.

### Dual luciferase assay

For the construction of the reporter plasmid, 2.5Kb of sequence upstream of the start codon of *TCP5* was first amplified from Col-0 genomic DNA and cloned with KpnI/PstI sites into the pGREEN800IILUC vector. For effector plasmids, the pDONR-RBE described above was clone into p2GW7 using the Gateway system (Invitrogen). GAL4-BDB- p2GW7 was described previously [62].

Transient expression assays were performed with *Arabidopsis* protoplasts as described [63]. Approximately 2-week old leaves were cut into strips by razor blades and submerged in 0.5 M mannitol solution. The leaves were then transferred to an enzyme containing solution (0.4 M mannitol, 20 mM KCl, 20 mM MES, pH 5.7, 0.015 g/mL Cellulose, 0.02 g/mL Macerozyme, 10 mM CaCl_2_, 0.35 mL/mL 2-Mercaptoethanol, and 1mg/mL BSA) and were incubated for 16h with gentle shaking at 50 rpm at room temperature. The protoplast was filtered out through a mesh with pore size of 150 mm. Protoplasts were then washed twice with W5 solution (154 mM NaCl, 125 mM CaCl_2_, 5 mM KCl, 2 mM MES (pH 5.7)), and then resuspended in MMG solution (0.4 M mannitol, 15 mM MgCl_2_, 4 mM MES (pH 5.7).

For each transformation, 5 μg of reporter plasmid and 5 μg of effector plasmid were used. The activities of LUC and REN were quantified after 13 hours incubation in the dark using the Dual Luciferase Assay kit (Promega) according to the manufacturer’s instructions. The LUC activity of each transformation was normalized to the REN activity (LUC/REN) and then analyzed against the negative control BD-GAL4.

### ChIP assay

ChIP was performed as described previously [45,64,65] with slight modifications. Collected inflorescence tissues (1.5g) were ground in liquid nitrogen and fixed in 1% (v/v) formaldehyde in 37mL ChIP Extraction Buffer 1 for 10 min. 2 mL of 2 M glycine was then added to quench the cross-linking for 5 min. The solution was filtered through layers of Miracloth (EMD Millipore) and then the nuclei were pelleted. The pellet was resuspended in Extraction Buffer 2. Nuclei and debris were pelleted through centrifugation (12000g, 10min, 4°C) twice and resuspended in 600 mL Extraction Buffer 3 followed by an overlay of 600 mL Extraction Buffer 3. The chromatin pellet was resuspended in 300 mL lysis buffer after centrifugation (16000g, 1h, 4°C). Chromatin was sonicated using a Bioruptor (Diagenode) for 20min (30s ON, 30s OFF, high level) to yield DNA fragments of 200-1000bp in length. The chromatin was precleared with Dynal Protein A beads (Invitrogen, 10004D) (1:20) for 1h then incubated with antibody (1:200 dilution) overnight. The immune complex was collected by Dynal Protein A beads (1:20 dilution) and then washed after 2h incubation. The eluted immune complex and the input sample were recovered by reverse-crosslinking with RNase treatment and proteinase K treatment. The DNA was purified by the ChIP DNA Clean & Concentrator (Zymo Research). Purified DNA was analyzed by qPCR analysis using the iTaq Universal SYBR Green Supermix (Bio-Rad) and Bio-Rad CFX96 real-time PCR detection system, with three technical replicates. The final relative enrichment was calculated by normalizing to the percent input. Two to four biological replicates were carried out for each experiment. The following antibodies were used for the ChIP-qPCR: anti-H3K9ac (07–352, Millipore), anti-H3K27me3 (07–449, Millipore), anti-RNAPII-CTD (ab26721, Abcam), anti-Myc (C3956, Millipore) and anti-HA (ab9110, Abcam). The primers used for ChIP are listed in S4 Table.

### FAIRE-qPCR

FAIRE was performed as described previously [37,52] with minor modifications. *35S*::*GR-RBE* inflorescence tissues were treated with 10μM dexamethasone or mock solution for 16h. The unopened floral buds from stage 1 to stage 12, along with the inflorescence meristem were collected and flash frozen in liquid nitrogen. For each trial, 0.5g tissues were ground and fixed with 1% formaldehyde (v/v) under vacuum for 8 min, replaced by 125mM Gly for 5 min on ice. For the UNFAIRE sample as control, 0.3g tissues were ground and processed without fixation. Chromatin was isolated and sonicated into fragments around 200 bp~700 bp as for ChIP. One volume of phenol: chloroform: isoamyl alcohol (25: 24: 1) was added to purify DNA. The qPCR was performed in three technical replicates using the SYBR Green PCR Master Mix (BioRad) reaction system. Four biological replicates and three technical replicates were carried for this experiment. Primers used for FAIRE-qPCR are listed in S4 Table.

## Supporting information

S1 Fig RBE interacts with TPR proteins *in planta*. Bimolecular fluorescence complementation assay (BiFC) to detect reconstitution of YFP fluorescence. YFP fluorescence shows interaction between RBE and TPR2 (top row), TPR3 (middle row) or TPR4 (bottom row) in the nuclei. Position of nuclei detected by fluorescence of H2B-mCherry. Panels (left to right): YFP; H2B-mCherry; merged. Scale bars 50 um.
(PDF)

S2 Fig RBE physically interacts with LHP1 via the EAR motif in Y2H assays. (A) Diagram of native RBE protein, RBE with point mutations in the EAR motif (mRBE) and RBE with an EAR motif-based artificial transcriptional repression domain [28] (mRBE-SRDX). (B) Yeast two-hybrid assay between RBE, mRBE or mRBE-SRDX with LHP1.
(PDF)

S3 Fig RBE interacts with LHP1 via the EAR motif *in planta*. Bimolecular fluorescence complementation assay (BiFC) to detect reconstitution of YFP fluorescence. YFP fluorescence shows interaction between RBE and LHP1 in the nuclei. Position of nuclei detected by fluorescence of H2B-mCherry. Panels (left to right): YFP; H2B-mCherry; merged. Scale bars 50 um.
(PDF)

S4 Fig RBE interacts with LDL1 via the EAR motif *in vivo*. Bimolecular fluorescence complementation assay (BiFC) to detect reconstitution of YFP fluorescence. YFP fluorescence shows interaction between RBE and LDL1 in the nuclei. Position of nuclei detected by fluorescence of H2B-mCherry. Panels (left to right): YFP; H2B-mCherry; merged. Scale bars 50 um.
(PDF)

S5 Fig OX-RBE lines with ectopic expression display significant difference in leaves. (A) qRT-PCR data of *RBE* variants in inflorescences of T1 transgenic overexpression lines. ACT2 served as the internal control. (B). The typical flowers are dissected from abovementioned transgenic overexpression lines. Scale bar, 1 cm. (C) Leaf phenotypes of abovementioned transgenic overexpression lines.
(PDF)

S6 Fig ChIP experiment to test HDA19 binding activity. ChIP assays using *35S*::*GR-RBE 35S*::*10xMyc-HDA19* seedlings after 16h DEX treatment. *Mu-like* transposon served as the negative control and its value was set to 1. Error bars represent mean ± SD of three biological replicates.
(PDF)

S7 Fig *TCP5* transcription is significantly downregulated 16h after DEX treatment. Relative levels of *TCP5* expression as assessed by qRT-PCR in Mock and DEX treated unopen buds. *Tipl41-like* served as internal control. Error bars represent mean ± SD of three biological replicates. *t*-test, **P< 0.01, *P <0.05.
(PDF)

S8 Fig Relative level of nuclear RBE-GR protein. Band intensities of each sample quantified by Image J were normalized relative to total histone H3 loading controls. Error bars represent mean ± SD of five biological replicates. *t*-test, **P< 0.01, *P <0.05.
(PDF)

S1 Table Primers used for genotyping. (DOCX)

S2 Table Primers used for yeast two hybrid. (DOCX)

S3 Table Primers used for qRT-PCR. (DOCX)

S4 Table Primers used for ChIP-qPCR and FAIRE-qPCR. (DOCX)
