# Supplementary figures and images for: An epigenetic timer regulates the transition from cell division to cell expansion during Arabidopsis petal organogenesis
Source: PLoS Genet. 2024 Mar 5;20(3):e1011203. doi: 10.1371/journal.pgen.1011203 (PMC10942257; doi:10.1371/journal.pgen.1011203)

S1 Fig

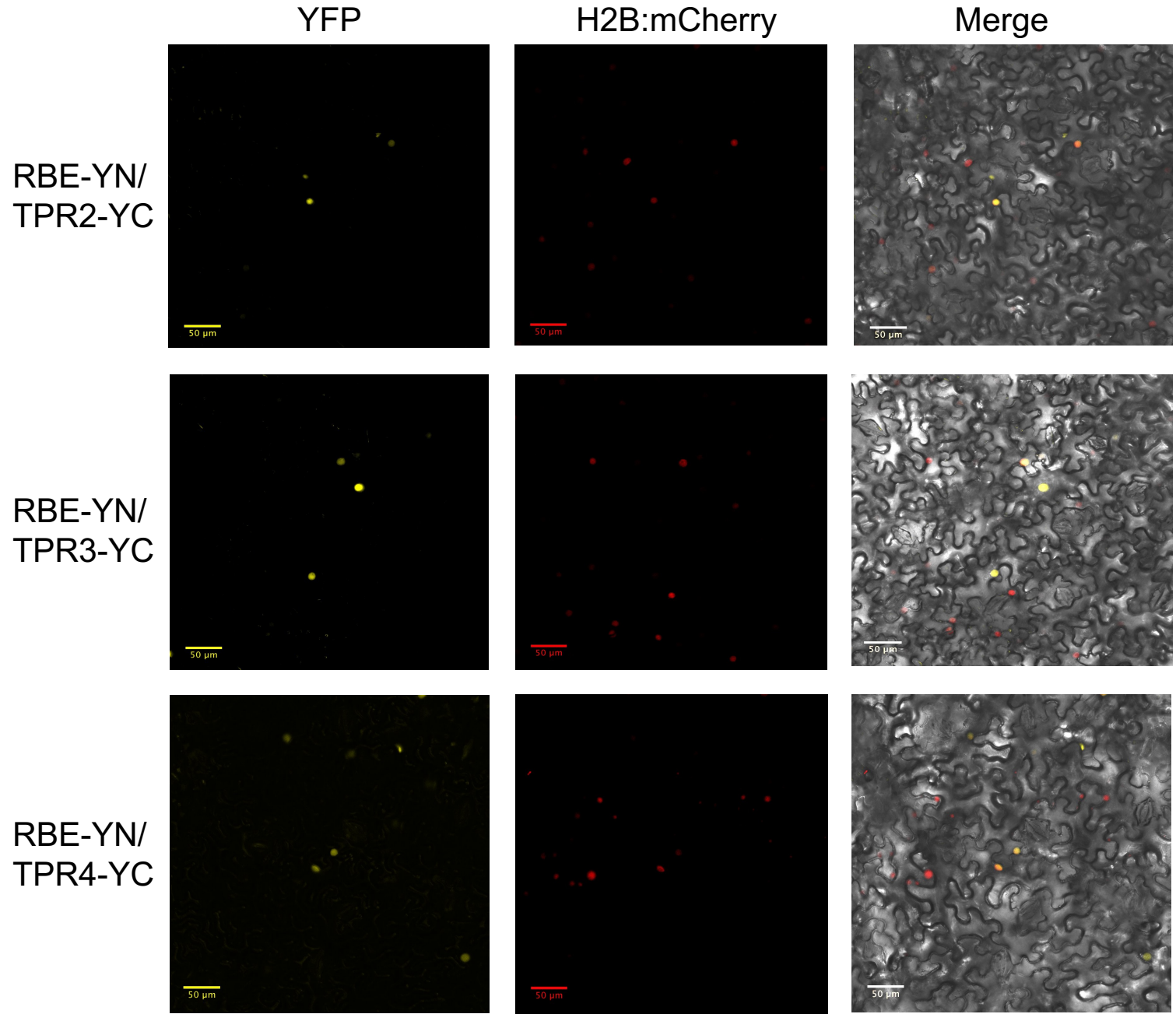

Supplement: S1 Fig — Bimolecular fluorescence complementation assay (BiFC) to detect reconstitution of YFP fluorescence. YFP fluorescence shows interaction between RBE and TPR2 (top row), TPR3 (middle row) or TPR4 (bottom row) in the nuclei. Position of nuclei detected by fluorescence of H2B-mCherry. Panels (left to right): YFP; H2B-mCherry; merged. Scale bars 50 um. (PDF) [file pgen.1011203.s001.pdf]

S2 Fig

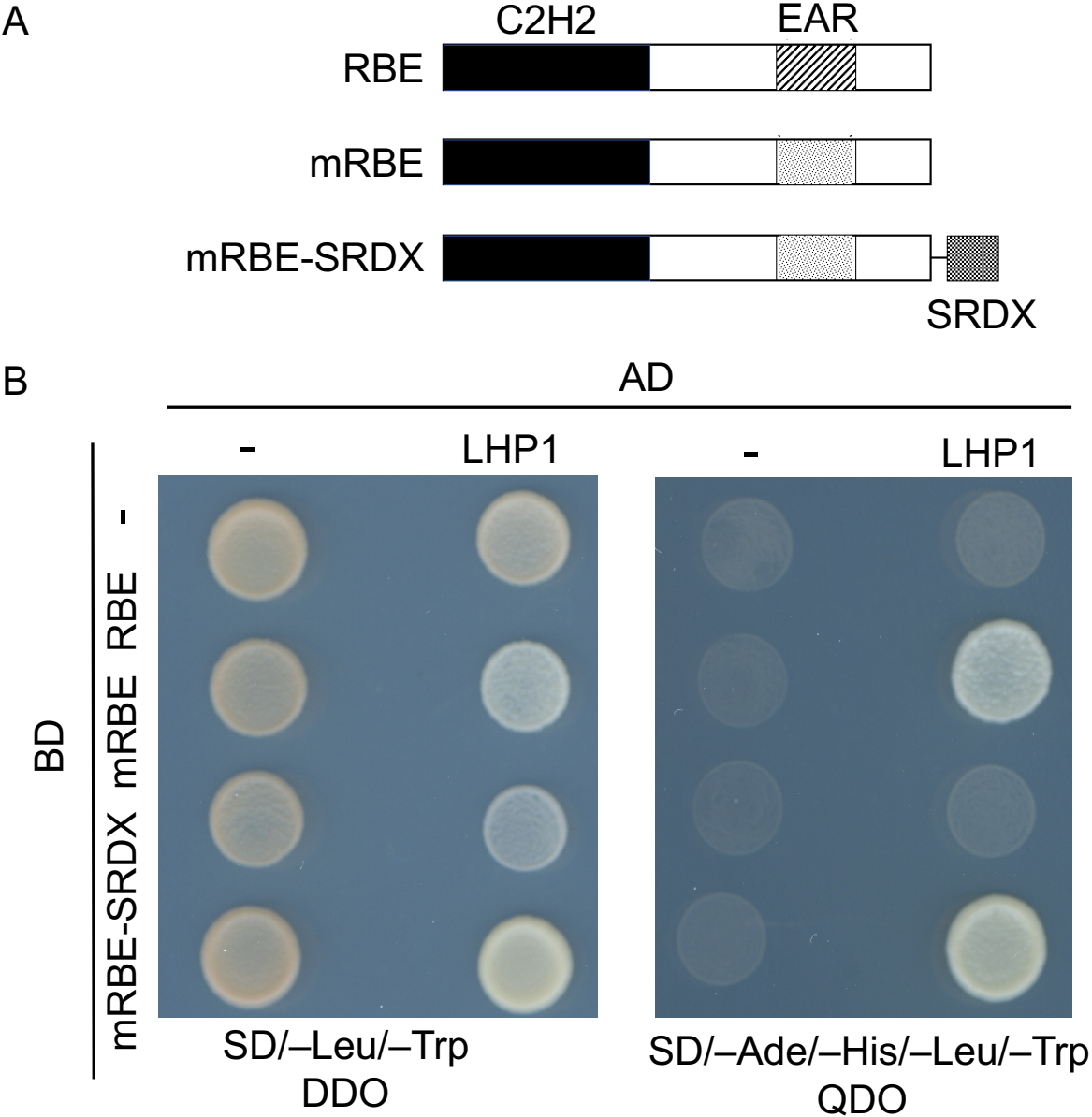

Supplement: S2 Fig — (A) Diagram of native RBE protein, RBE with point mutations in the EAR motif (mRBE) and RBE with an EAR motif-based artificial transcriptional repression domain [28] (mRBE-SRDX). (B) Yeast two-hybrid assay between RBE, mRBE or mRBE-SRDX with LHP1. (PDF) [file pgen.1011203.s002.pdf]

S3 Fig

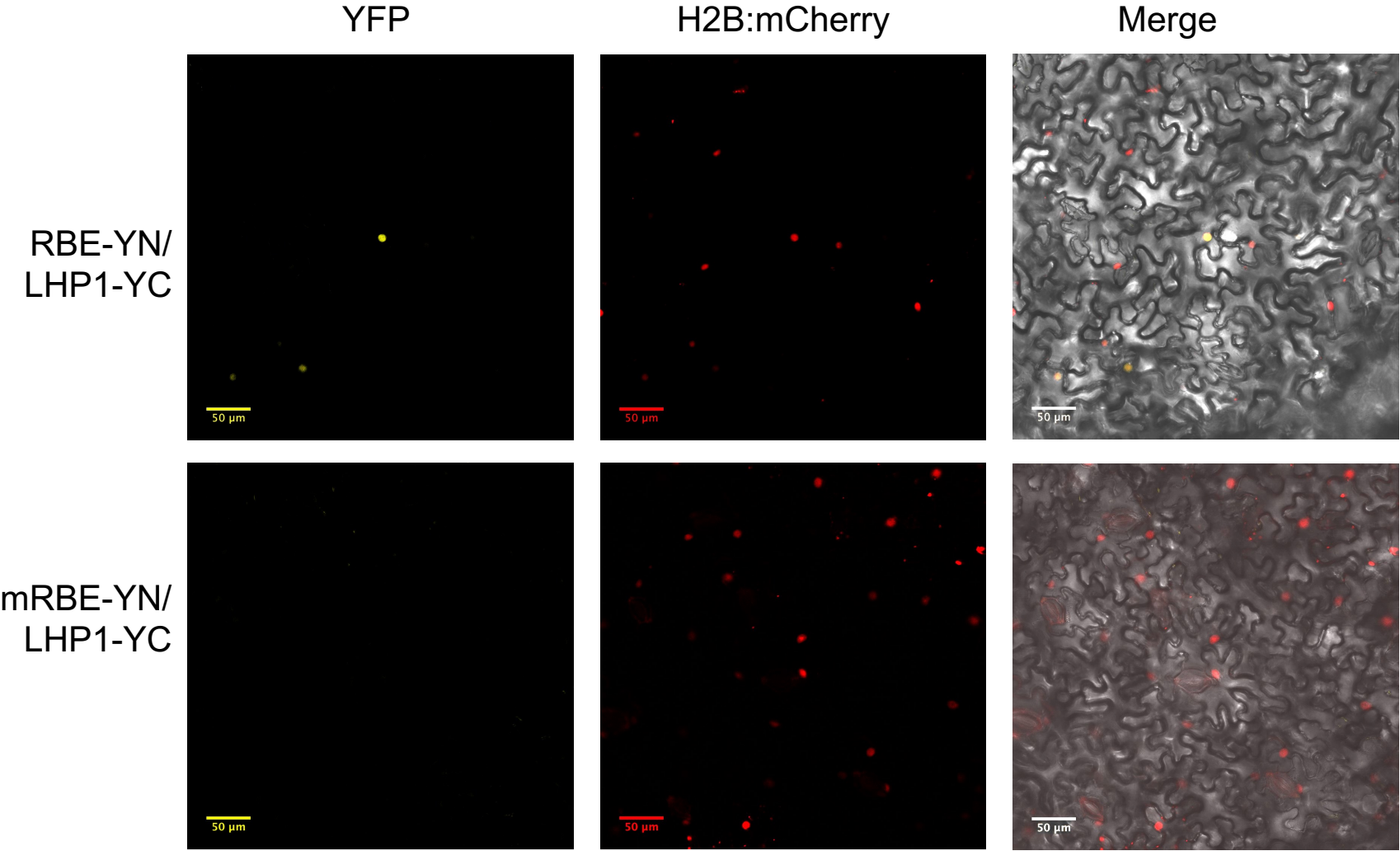

Supplement: S3 Fig — Bimolecular fluorescence complementation assay (BiFC) to detect reconstitution of YFP fluorescence. YFP fluorescence shows interaction between RBE and LHP1 in the nuclei. Position of nuclei detected by fluorescence of H2B-mCherry. Panels (left to right): YFP; H2B-mCherry; merged. Scale bars 50 um. (PDF) [file pgen.1011203.s003.pdf]

S4 Fig

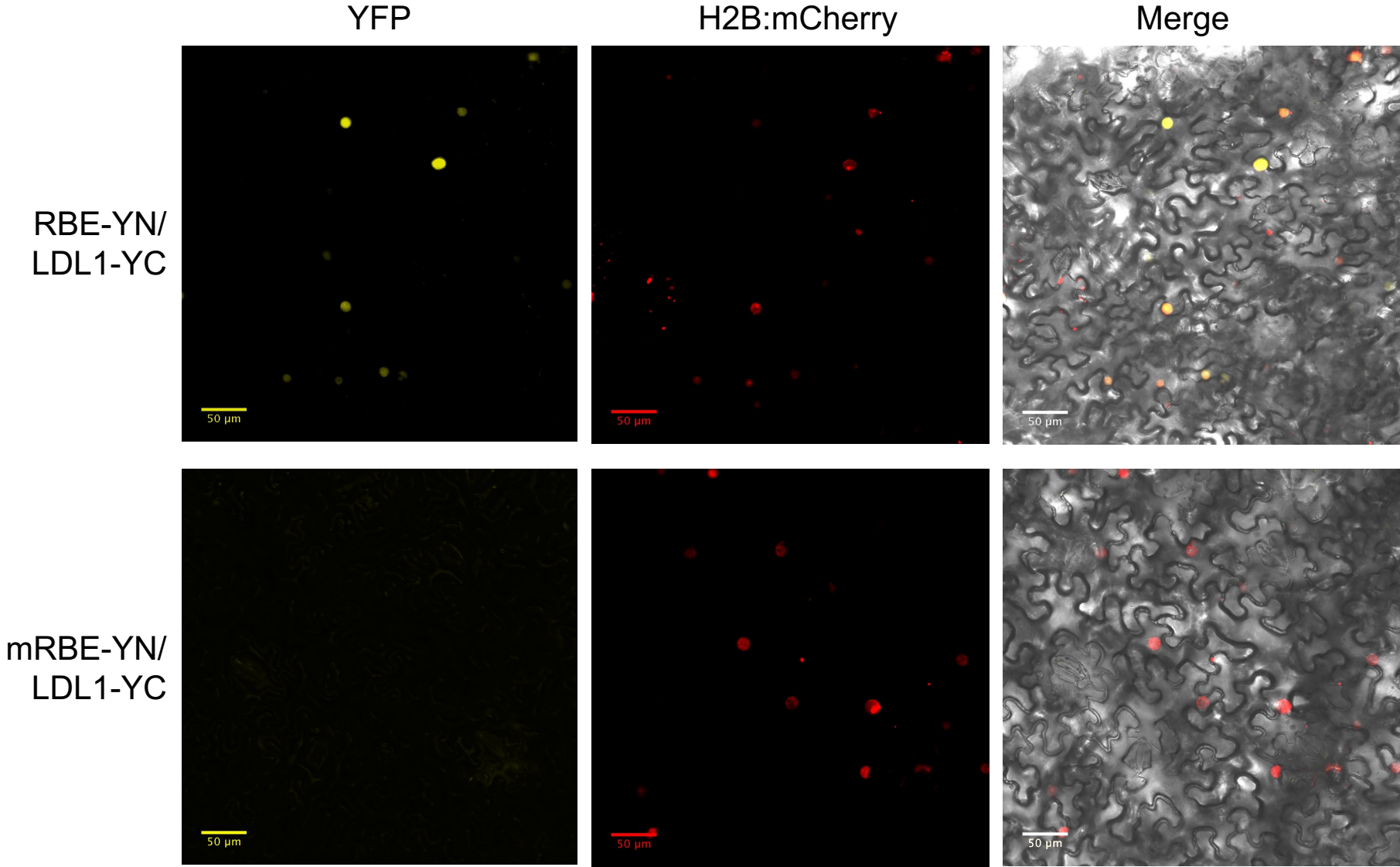

Supplement: S4 Fig — Bimolecular fluorescence complementation assay (BiFC) to detect reconstitution of YFP fluorescence. YFP fluorescence shows interaction between RBE and LDL1 in the nuclei. Position of nuclei detected by fluorescence of H2B-mCherry. Panels (left to right): YFP; H2B-mCherry; merged. Scale bars 50 um. (PDF) [file pgen.1011203.s004.pdf]

S5 Fig

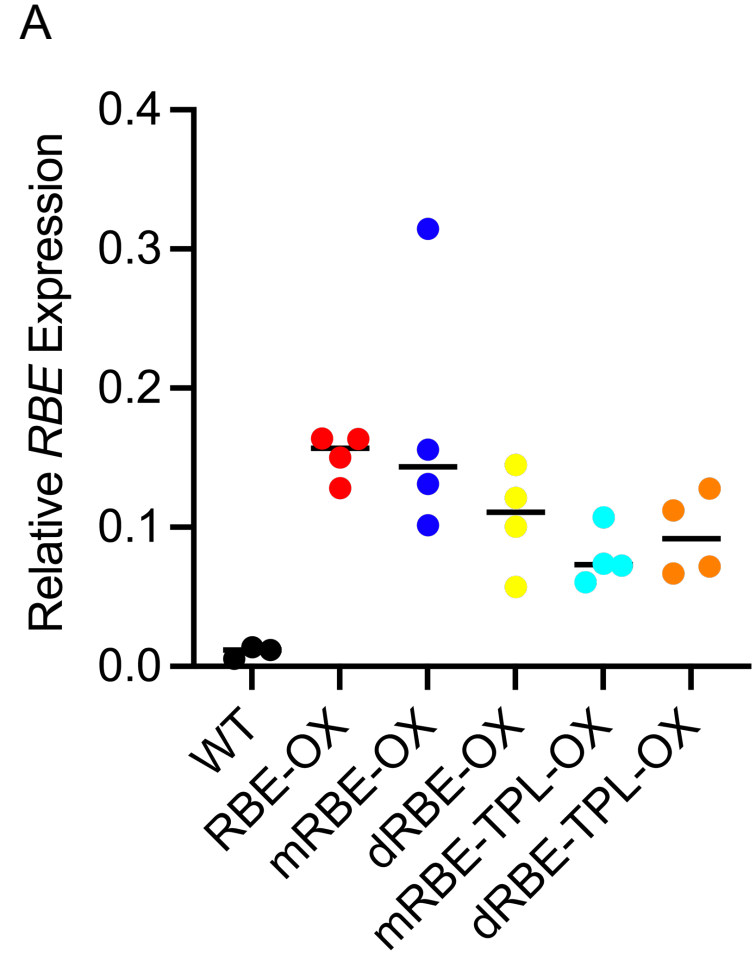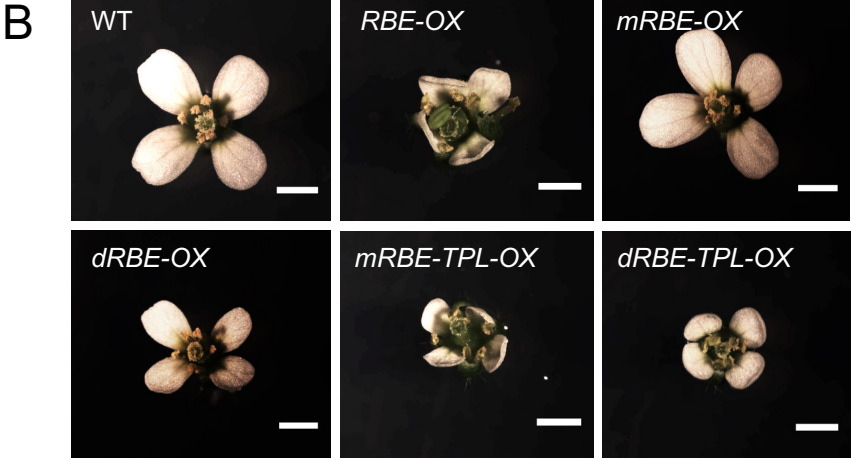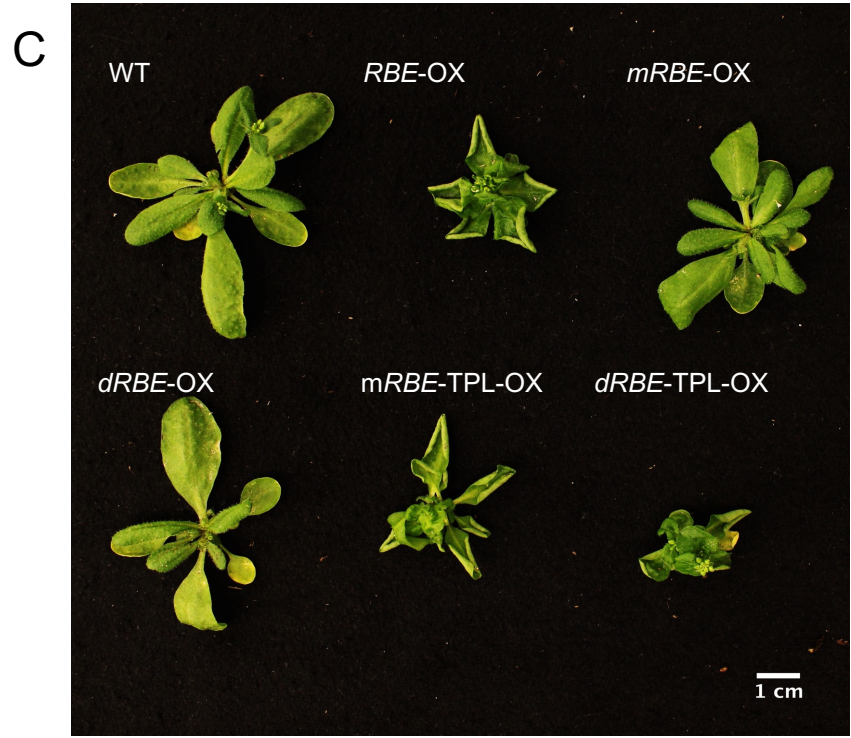

Supplement: S5 Fig — (A) qRT-PCR data of RBE variants in inflorescences of T1 transgenic overexpression lines. ACT2 served as the internal control. (B). The typical flowers are dissected from abovementioned transgenic overexpression lines. Scale bar, 1 cm. (C) Leaf phenotypes of abovementioned transgenic overexpression lines. (PDF) [file pgen.1011203.s005.pdf]

S6 Fig

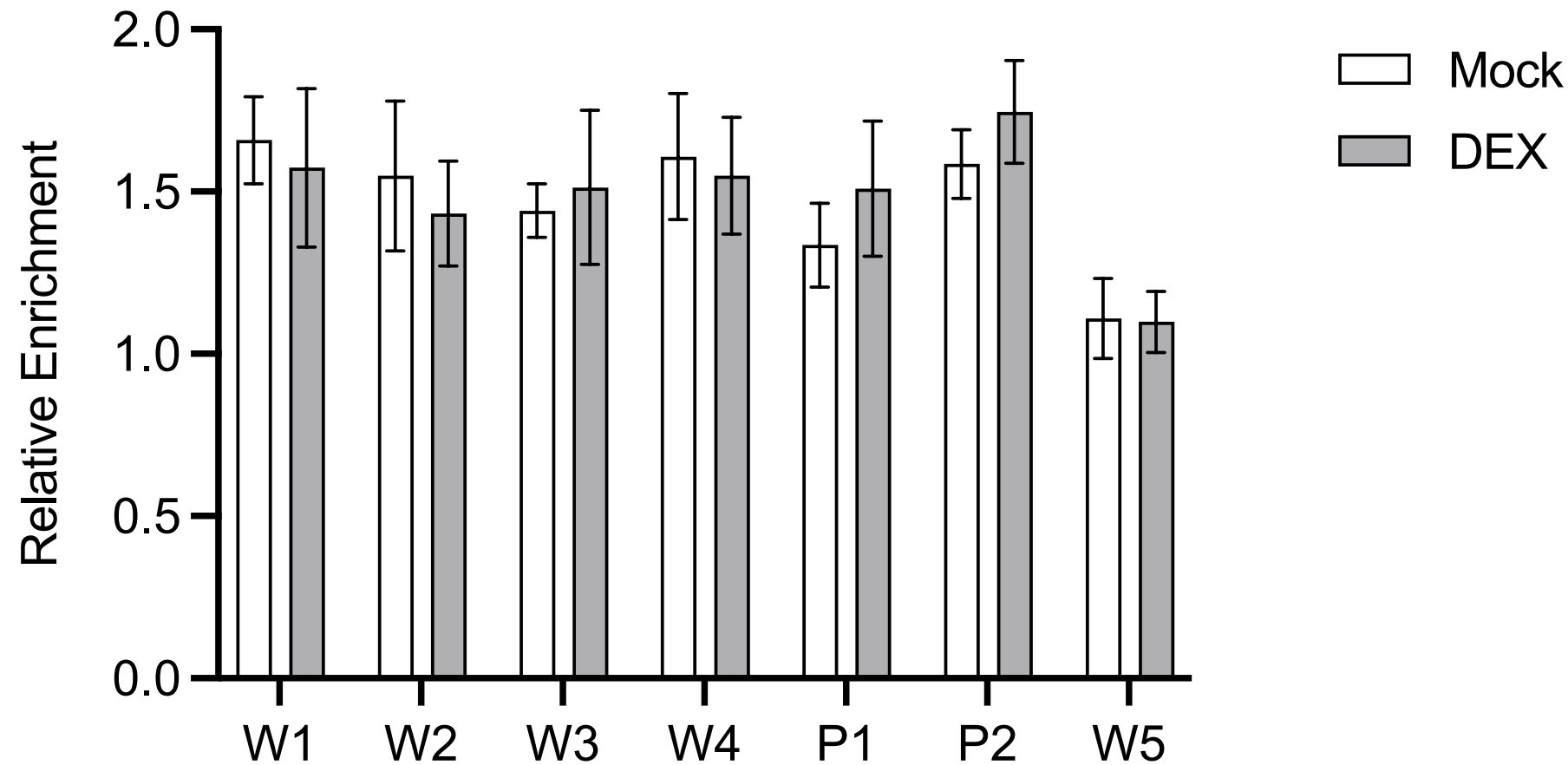

Supplement: S6 Fig — ChIP assays using 35S::GR-RBE 35S::10xMyc-HDA19 seedlings after 16h DEX treatment. Mu-like transposon served as the negative control and its value was set to 1. Error bars represent mean ± SD of three biological replicates. (PDF) [file pgen.1011203.s006.pdf]

S7 Fig

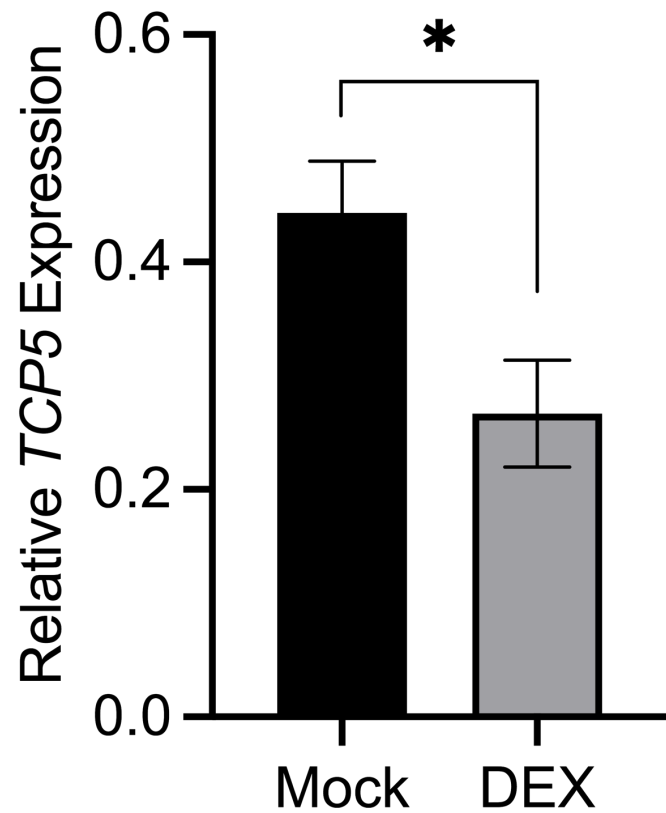

Supplement: S7 Fig — Relative levels of TCP5 expression as assessed by qRT-PCR in Mock and DEX treated unopen buds. Tipl41-like served as internal control. Error bars represent mean ± SD of three biological replicates. t-test, **P< 0.01, *P <0.05. (PDF) [file pgen.1011203.s007.pdf]

S8 Fig

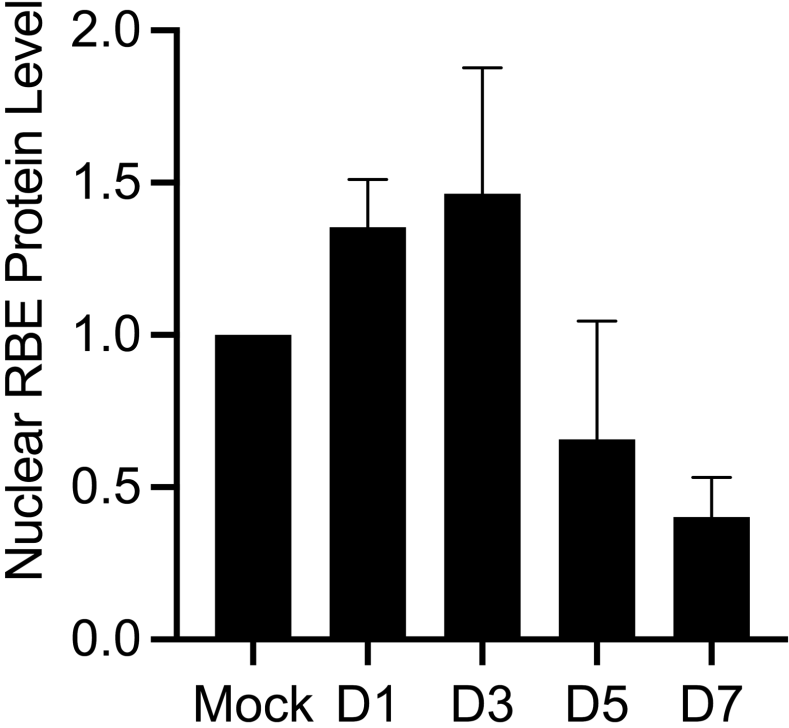

Supplement: S8 Fig — Band intensities of each sample quantified by Image J were normalized relative to total histone H3 loading controls. Error bars represent mean ± SD of five biological replicates. t-test, **P< 0.01, *P <0.05. (PDF) [file pgen.1011203.s008.pdf]
