# Supplementary material for: An epigenetic timer regulates the transition from cell division to cell expansion during Arabidopsis petal organogenesis
Source: PLoS Genet. 2024 Mar 5;20(3):e1011203. doi: 10.1371/journal.pgen.1011203 (PMC10942257; doi:10.1371/journal.pgen.1011203)
Supplement: S1 Table — (DOCX) [file pgen.1011203.s009.docx]

**S1 Table. Primers used for genotyping.**

| **Mutant** | **Primer name** | **Primer sequence (5′-to-3′)** | **Other** |
| --- | --- | --- | --- |
| *rbe-1* | RBE1GF | AGGTCTTACTCATGCAGCTTTTGC | DdeI |
|  | RBE1GR | AGAACCTTTGATCCCACATCAAGA | DdeI |
| *tpl-1* | tpl-1F | ATGTAGTGTCCAAAGCCTTTGT | AlwI |
|  | tpl-1R | TTAAGCTGCGAGTTATGCAGTA | AlwI |
| *hda19-1* | hda19-1F | GAGCTATCATCTGTTATTCAAGCCC | Span T-DNA |
|  | hda19-1R | GCAAGAAATTAGAAGCTCCGAGTC | Span T-DNA |
