# Supplementary material for: An epigenetic timer regulates the transition from cell division to cell expansion during Arabidopsis petal organogenesis
Source: PLoS Genet. 2024 Mar 5;20(3):e1011203. doi: 10.1371/journal.pgen.1011203 (PMC10942257; doi:10.1371/journal.pgen.1011203)
Supplement: S2 Table — (DOCX) [file pgen.1011203.s010.docx]

**S2 Table. Primers used for yeast two hybrid.**

| **Gene name** | **Primer name** | **Primer sequence (5′-to-3′)** |
| --- | --- | --- |
| RBE | RBEF | GGGGACAAGTTTGTACAAAAAAGCAGGCTTCATGATGGATAGAGGAGAATGCTTG |
|  | RBER | GGGGACCACTTTGTACAAGAAAGCTGGGTCCTAGTTAACCTTAGGCGGATCAG |
| TPL | TPLF | GGGGACAAGTTTGTACAAAAAAGCAGGCTTCATGTCTTCTCTTAGTAGAGAGCTC |
|  | TPLR | GGGGACCACTTTGTACAAGAAAGCTGGGTCTCATCTCTGAGGCTGATCAGA |
| HDA19 | HDA19F | GGGGACAAGTTTGTACAAAAAAGCAGGCTTCATGGATACTGGCGGCAATTC |
|  | HDA19R | GGGGACCACTTTGTACAAGAAAGCTGGGTCTTATGTTTTAGGAGGAAACGCCT |
| TPR2 | TPR2F | GGGGACAAGTTTGTACAAAAAAGCAGGCTTCATGTCGTCTTTGAGCAGAGAG |
|  | TPR2R | GGGGACCACTTTGTACAAGAAAGCTGGGTCTTACCTTTGAATCTGATCCGAAC |
| TPR3 | TPR3F | GGGGACAAGTTTGTACAAAAAAGCAGGCTTCATGTCGTCGTTGAGTCGAGAG |
|  | TPR3R | GGGGACCACTTTGTACAAGAAAGCTGGGTCTCATCTTTGTAACTGTTCTGGAG |
| TPR4 | TPR4F | GGGGACAAGTTTGTACAAAAAAGCAGGCTTCATGTCGTCACTCAGCAGAG |
|  | TPR4R | GGGGACCACTTTGTACAAGAAAGCTGGGTCCTACGAATCACTCGGTTGTTGAT |
| LHP1 | LHP1F | GGGGACAAGTTTGTACAAAAAAGCAGGCTTCATGAAAGGGGCAAGTGGTG |
|  | LHP1R | GGGGACCACTTTGTACAAGAAAGCTGGGTCTTAAGGCGTTCGATTGTACTTGA |
| LDL1 | LDL1F | GGGGACAAGTTTGTACAAAAAAGCAGGCTTCATGTCAACAGAGACTAAAGAAAC |
|  | LDL1R | GGGGACCACTTTGTACAAGAAAGCTGGGTCCTAATCAAAGATCTGTCGATTC |
