# Supplementary material for: An epigenetic timer regulates the transition from cell division to cell expansion during Arabidopsis petal organogenesis
Source: PLoS Genet. 2024 Mar 5;20(3):e1011203. doi: 10.1371/journal.pgen.1011203 (PMC10942257; doi:10.1371/journal.pgen.1011203)
Supplement: S3 Table — (DOCX) [file pgen.1011203.s011.docx]

**S3 Table. Primers used for qRT-PCR.**

| **Gene** | **Primer Name** | **Primer sequence (5′-to-3′)** |
| --- | --- | --- |
| RBE | F | ACGGAACCAAGAGGGAGATAA |
|  | R | ACTCAGTAGACCGACCGATAAG |
| TCP5 | F | TCCCGACATACCCTTCGTTT |
|  | R | TCATGATTCGAGCTCATCAA |
| Tip41-like | F | GTGAAAACTGTTGGAGAGAAGCAA |
|  | R | TCAACTGGATACCCTTTCGCA |
| ACT2 | F | GGTAACATTGTGCTCAGTGGTGG |
|  | R | AACGACCTTAATCTTCATGCTGC |
