# Supplementary material for: An epigenetic timer regulates the transition from cell division to cell expansion during Arabidopsis petal organogenesis
Source: PLoS Genet. 2024 Mar 5;20(3):e1011203. doi: 10.1371/journal.pgen.1011203 (PMC10942257; doi:10.1371/journal.pgen.1011203)
Supplement: S4 Table — (DOCX) [file pgen.1011203.s012.docx]

**S4 Table. Primers used for ChIP-qPCR and FAIRE-qPCR.**

| **Gene** | **Primer name** | **Primer sequence (5′-to-3′)** |
| --- | --- | --- |
| TCP5 | P1F | GTTTCAGATCTGTTCTTTACACCAA |
|  | P1R | CTTGCTTTGCTTGAATCTCCTC |
|  | P2F | CAACAACAACAGCCGAGTTC |
|  | P2R | TGTCTTTGCCACCGAATGT |
|  | P3F | GTTGTCCGTACCTACAGCTATTC |
|  | P3R | GGTAGCTTGTCTACGTCATCTTT |
|  | P4F | GGAACCAATCTCGGGTTCTT |
|  | P4R | GGGTCGTAGTTGTATCGGTTAAT |
|  | P5F | CCTTCGTTTCTTGGAGCTTCT |
|  | P5R | TGCTGCGATGCGGTATTT |
|  | W1F | AAAAGCAGTATTCCCAACAGTTTGA |
|  | W1R | ACGAAGCAACTTGTTGAAACCCTA |
|  | W2F | TTTCACAGTTCAAAGTTCAAACCAC |
|  | W2R | AAGGTCATTTAAGAGAAAGTGTACGG |
|  | W3F | AAGATTTATGTGACTGTGAGTGTAACGA |
|  | W3R | GCAGAAGGACAAAAGAGGAGCAAT |
|  | W4F | GCAAAGACAGACACAGCAAAG |
|  | W4R | TCTTGAAGGTCGTAGAGTTGAATAG |
|  | W5F | TGCGTATTGTTGGATTGGTTTATG |
|  | W5R | TCTTGAAGGTCGTAGAGTTGAATAG |
| TA3 | TA3F | CTGCGTGGAAGTCTGTCAAA |
|  | TA3R | CTATGCCACAGGGCAGTTTT |
| EIF4 | EIF4F | GCTGAGTTGGGAGATCGAAGTT |
|  | EIF4R | TCTGGTTCCTCCCGTGTTCT |
| MU-like | MUF | GATTTACAAGGAATCTGTTGGTGGT |
|  | MUR | CATAACATAGGTTTAGAGCATCTGC |
